# Using clustered data to develop biomass allometric models: The consequences of ignoring the clustered data structure

**DOI:** 10.1371/journal.pone.0200123

**Published:** 2018-08-02

**Authors:** Ioan Dutcă, Petru Tudor Stăncioiu, Ioan Vasile Abrudan, Florin Ioraș

**Affiliations:** 1 Faculty of Silviculture and Forest Engineering, Transilvania University of Brasov, Brasov, Romania; 2 Buckinghamshire New University, High Wycombe, United Kingdom; Texas A&M University, UNITED STATES

## Abstract

This paper investigates the consequences of ignoring the clustered data structure on allometric models. Clustered data, in the form of multiple trees sampled from multiple forest stands is commonly used to develop biomass allometric models. Of 102 reviewed papers published between 2012 and 2016 that reported biomass allometric models, 84 (82%) have used a clustered sampling design. However, in as many as 80% of these, the clustered data structure was ignored, potentially violating the independence assumption in ordinary least squares methods. The consequences of ignoring clustered data structure were empirically validated using two clustered biomass datasets (of 110 and 220 trees, with the cluster size of 5 and 10 trees respectively). We showed that when Intraclass Correlation Coefficient (ICC) was higher than zero, ignoring the clustered data structure returned underestimated standard errors, affecting further the confidence interval and *t*-test results. The underestimation level depended on ICC (which shows the variance proportion that was caused by the forest stand) and on cluster size (the number of trees sampled from one forest stand). We also showed that using first-order autocorrelation tests, such as the traditional Durbin-Watson statistic, to detect the autocorrelation due to clustered structure could be misleading as the test may show lack of autocorrelation even though ICC is different from zero. In conclusion, when ICC is higher than zero, ignoring the clustered data structure yields over-confident biomass predictions (due to underestimated confidence interval) and/or incorrect research conclusions (due to overestimated evidence against null hypothesis in *t*-test). Therefore, using a modelling approach that accounts for the hierarchical structure of the data is highly recommended when any form of clustering can be identified, even if the autocorrelation is not significant.

## 1. Introduction

Estimating carbon accumulation in forests, with great accuracy and precision, represents one of the major challenges that the international scientific community is facing today, in the context of climate change. However, regardless of how accurate and precise these estimations are, they have to be robust. Robustness does not imply low uncertainty, but assumes that all uncertainty is known and transparently presented. The uncertainty is an essential component that helps policy makers understand how much these estimations can be trusted in order to make correct decisions regarding effective policies concerning climate change mitigation [1].

Quantifying and reducing the uncertainty in GHG estimations in forestry sector is becoming increasingly important, especially in the context of result-based payments in REDD+ programme (i.e. a mechanism for reducing emissions from deforestation and forest degradation, under the United Nations Framework Convention on Climate Change), and of emission reduction commitments under Kyoto Protocol. Estimating and reporting uncertainty under IPCC (Intergovernmental Panel on Climate Change) guidelines [2], is mandatory for assessing the performance in implementation of all these land related activities for emission reduction.

Despite the recent advances in remote-sensing based carbon monitoring [3], biomass allometric models are widely used to estimate forest biomass [4,5] or to calibrate the remote-sensing based methods [6]. The allometric models use easy-to-measure characteristics (e.g. diameter, height) to predict tree biomass, and subsequently the carbon sequestered in biomass. In forestry practice empirical allometric models are often used, since they offer more accurate biomass prediction compared to theoretical models [7,8]. The empirical models are developed based on destructive sampling of trees, which involves the measurement of biomass (including all vegetative organs of the tree, both above and below ground) and of dendrometric characteristics of the standing tree (e.g. diameter at breast height, root collar diameter, height, crown diameter). The most common method of data analysis consists of logarithmic transformation of the variables, followed by classic linear regression analysis (Ordinary Least Squares, OLS) and a back-transformation [9]. Although logarithmic transformation has been criticized lately, the goodness of fit for the proposed alternative (i.e. non-linear approach) depends on the type of error distribution. A non-linear approach was shown to be better for additive, homoscedastic normal error distributions, whereas logarithmic transformation was shown to give better results for multiplicative lognormal errors, which occur more frequently in allometric models [10]. To fully trust the results, several assumptions (i.e. independence, linearity, normality and homogeneity of variance) should be fulfilled by the OLS regression [11]. However, the independence of observations is one of the most important and one of the most widely-ignored regression assumptions [12].

The range of covariate (i.e. diameter or height) in allometric models is recommended to be large [13], with a suggested minimum range of one order of magnitude. However, the range of covariate is often limited within relatively uniform forest stands, especially in even-aged, planted forests where the trees have relatively similar sizes. Consequently, it is often necessary to sample trees from more than one forest stand, resulting in clustered datasets (more generally called ‘nested’ datasets). The issue with clustered data resides in the violation of the independence assumption [14], when OLS methods are used. Due to similarities in genotype, environmental conditions and stand competition, the trees from the same stand (especially from regular plantations) tend to be more similar to each other than trees from other forest stands. Thus, when the data is clustered, the variance is produced both by the variability of the trees within and between forest stands. If the variance produced by forest stands (between-stands variance) is different from zero, the individual trees do not bring the full amount of information, as OLS regression assumes for fully independent observations. Instead, the new information each observation (i.e. tree) brings to the model, becomes weaker as the proportion of between-stands variance gets larger. As a result, when OLS methods are used with clustered data the standard errors are biased [15].

This work focuses on the following research questions: (i) How frequently is clustered data structure ignored in biomass data analysis? (ii) What are the consequences of ignoring the clustered data structure in allometric models? (iii) How effective is Durbin-Watson test in detecting autocorrelation resulting from clustering?

## 2. Materials and methods

### 2.1. Review of published research

We assessed whether using clustered data to develop allometric equations and whether ignoring the clustered data structure were common in published allometric models. A total of 102 papers published in the last five years (January 2012 to December 2016) that reported biomass allometric equations were reviewed. Using specific keywords, the papers were searched online in February 2017. The sampling design of each paper was evaluated, to check whether the sampling design was clustered or not. The dataset was considered clustered (or nested) if data was collected from more than one forest stand (or location) with more than one tree sampled in at least one location. The dataset was also considered not independent, if biomass data for different tree species was pooled together to develop a multispecies model. The sampling design was considered independent if there was no or unclear information about clustered sampling. Besides the sampling design, the use, or non-use, of statistical techniques that include the clustering (e.g. multilevel models) was checked, for those papers suspected of independence violation.

### 2.2. Theoretical modelling framework

Here we present the general modelling framework for the linear model (which ignores the clustered data structure) and the multilevel model (which addresses the clustered data structure). Furthermore, the consequences of ignoring the clustered data structure (when using linear models) are derived from this framework.

#### 2.2.1. Ignoring the clustered data structure: Linear model (LM)

Ordinary least squares linear model (LM) assumes that all observations are independent:

$$y_{i}=\alpha +\beta x_{i}+\varepsilon_{i}$$ (1)

where *y* is the dependent variable (e.g. tree biomass); *x* is the independent variable (e.g. tree diameter or height); *α* is the intercept; *β* is the slope; *ε* is the error term. Therefore, the clustered data structure is ignored, as the model cannot incorporate the dependency within the data [15].

#### 2.2.2. Addressing the clustered data structure: Multilevel model (MLM)

The multilevel model, also called the hierarchical or mixed-effect model, can incorporate the variance produced by forest stand, producing adjusted standard errors. It was also shown to produce also correct type I error rates [16]. In multilevel analysis, the trees are referred to as level 1, whereas the cluster (plantation or forest stand) is referred to as level 2. The prediction is made at level 1 only (tree level). As the multilevel model provides different intercepts (one for each analysed forest stand), using them for biomass prediction in other forest stands makes little sense. Therefore, in this case, the role of level 2 is to allow the quantification of the noise generated by the forest stand [17], called also ‘nuisance effect’ [15]. Consequently, the best linear unbiased predictor (BLUP) is derived from all intercept values, the resulting multilevel model taking similar form to that of linear model, while accounting for correlation within the data structure:

Level 1:
  $$y_{ij}=\alpha +u_{j}+\beta x_{ij}+\varepsilon_{ij},\;for\;i=1,\ldots ,N,\;\varepsilon_{ij}\sim N(0,\sigma^{2})$$ (2)
Level 2:
  $$u_{j}\sim N(0,\tau^{2}),\;for\;j=1,\ldots ,J$$ (3)
  Where *α* is the best linear unbiased predictor (BLUP) of the intercept, based on restricted maximum likelihood method; *u*_*j*_ is the random part of the intercept, which assumes a normal distribution, with mean zero and variance *τ*^2^; *β* is the fixed slope for the population; *J* is the number of groups (forest stands); *N* is the total number of trees; *y*_*ij*_ is the biomass of the tree *i* from forest stand *j*; *x*_*ij*_ is the diameter (or height) of the tree *i* from forest stand *j*; *ε*_*ij*_ is the error of tree *i* from forest stand *j*. The error variance is assumed normal with mean zero and variance *σ*^2^; *u*_*j*_ and *ε*_*ij*_ are assumed to be mutually independent.

In multilevel models, the standard errors are adjusted by square root of design effect (*D*_*eff*_) [14]:

$$D_{eff}=1+(n-1)\times ICC$$ (4)

where *n* is the cluster size (number of trees sampled from one forest stand) and ICC is the Intraclass Correlation Coefficient. ICC shows the proportion of variance that is due to differences between clusters (level 2), out of total residual variance (level 1 and level 2):

$$ICC=\frac{\tau^{2}}{\tau^{2}+\sigma^{2}}$$ (5)

where: *τ*^2^ is the random variance that is attributed to between cluster variation (*τ* is random effect of the intercept); *σ*^2^ is the residual variance, caused by the difference between trees within forest stand (*σ* is residual random effect). ICC varies between 0 and 1. When ICC = 0, all variance is due to differences between individuals within clusters. When ICC = 1, all individuals within clusters are perfectly correlated and therefore, all variance of the model results from differences between clusters.

The design effect also shows the ratio between the actual number of observations and the effective number of observations [14]. The effective number of observations is a hypothetical value, which can be defined as the number of fully independent observations that would offer the same output as the non-independent observations (i.e. actual number of observations). If *n* = 1, the effective number of observations equals the actual number of observations, therefore the data is independent. The data is also considered independent when ICC = 0, regardless of cluster size. However, when both ICC > 0 and *n* > 1, the independence assumption is violated, and the effective number of observations becomes lower than the actual number of observations. When ICC = 1, the effective number of observations equals the number of clusters, as all trees within one cluster will bring identical information to the model. In contrast, even when ICC has reasonably low values, the effective number of observations can be very seriously affected, if *n* is large.

#### 2.2.3. The consequences of ignoring the clustered data structure

Because the trees are likely to be more similar inside a particular forest stand, the autocorrelation in allometric models is expected to be only positive. Nevertheless, positive autocorrelation of residuals produces underestimation of standard errors [18], since the number of effective observations is lower than the actual number of observations. The underestimation of standard errors (*SE*_*u*_) was defined as:

$${SE}_{u}=\frac{{SE}_{mlm}-{SE}_{lm}}{{SE}_{mlm}}\times 100$$ (6)

where *SE*_*mlm*_ is the standard error of the multilevel model parameters (intercept and slope), and *SE*_*lm*_ is the standard error of the linear model parameters. If the standard errors are weighted by square root of design effect, Eq 6 can be written as a function of *n* and ICC:

$${SE}_{u}=\frac{\sqrt{1+(n-1)\times ICC}-1}{\sqrt{1+(n-1)\times ICC}}\times 100$$ (7)

However, the standard errors are generally used to compute the confidence intervals and also the evidence against null hypothesis in t-test. The confidence intervals in logarithmic scale are underestimated by the same rate as standard errors (Eq 7), due to the proportionality between confidence interval and standard error. Instead, in null hypothesis tests of the slope (*t*-test), the slope estimate is divided by its standard error to obtain *t*-score. Therefore, the underestimation of standard errors produces an overestimation of evidence against null hypothesis in *t*-test (called *t*-score). The relative overestimation of *t*-score (*t*_*ovr*_), as resulted from ignoring the clustered data structure, was calculated based on the *t*-score of linear model (*t*_*lm*_) and the *t*-score of multilevel model (*t*_*mlm*_):

$$t_{ovr}=\frac{t_{lm}-t_{mlm}}{t_{mlm}}\times 100$$ (8)

Incorporating the squared root of design effect (Eq 4) into Eq 8, the overestimation of *t*-score, can be written as:

$$t_{ovr}=\frac{\beta_{lm}\sqrt{D_{eff}}-\beta_{mlm}}{\beta_{mlm}}\times 100$$ (9)

where *β*_*lm*_ is the slope resulted from linear model and *β*_*mlm*_ is the slope resulted from multilevel model. Under the assumption that the difference between *β*_*lm*_ and *β*_*mlm*_ tends to zero, Eq 9 becomes:

$$t_{ovr}=(\sqrt{1+(n-1)\times ICC}-1)\times 100$$ (10)

### 2.3. Empirical validation

The two theoretical models (Eqs 7 and 10) describe respectively the theoretical underestimation of standard errors (and of confidence interval) and the theoretical overestimation of evidence against null hypothesis in *t*-test, by ignoring the clustered data structure. These models were validated using the biomass data collected from 22 plantations of Norway spruce located in Eastern Carpathians of Romania (see S1 Appendix), for a total of 220 trees. In each plantation a 200 m^2^ sample plot was established to determine the tree of average size, based on diameter at collar height. From each plantation, ten trees with close dimensions to those of average size were destructively sampled and measured for biomass (for detailed biomass measurement method see [19]). For each tree, dried biomass of stem (ST), branches (BR), needles (ND) and roots (RT) were measured (in grams). Diameter at collar height (in mm) and tree height (in cm) were measured in situ before and respectively after tree felling. Access to plantations was granted by the National Forest Administration—ROMSILVA and two private forest districts: R.P.L.P. Kronstadt R.A. and O.S. Izvorul Somesului R.A.

The data produced was used to build two empirical biomass datasets:

- Dataset 1 (*n* = 5, *n* is the cluster size): this is a subset of the entire dataset produced. Five trees from those 10 sampled in each plantation were randomly selected. Therefore, the first dataset comprises 110 trees (22 plantations × 5 trees sampled from each plantation).
- Dataset 2 (*n* = 10): this included all 220 sampled trees (22 plantations × 10 trees sampled from each plantation).

The relationship between biomass and diameter (or height) is not linear, being widely accepted that this relationship takes a power function [20]. Therefore, adopting logarithmic transformation of variables is often done, in order to obtain a linear relationship between variables and to remove the heteroscedasticity. Transforming back from linear to ‘power’ form equation results in a bias [21]. However, this bias can be counteracted by using a correction factor [21,22], that is based on Residual Standard Error (*RSE*):

$$\lambda =e^{\left(\frac{{RSE}^{2}}{2}\right)}$$ (11)

The dependent variables were represented by biomass components: stem biomass (ST); branch biomass (BR); needle biomass (ND); root biomass (RT) and total tree biomass (TB) resulted by adding together all tree biomass components. The independent variables are root collar diameter (D) and height (H). All these variables were transformed using the natural logarithm (ln).

The observed underestimation of standard errors (and of confidence intervals) was calculated using Eq 6, for each dataset and for each model (10 models for each dataset, resulted from combinations of 5 biomass components and 2 predictors: TB = *f*(D); TB = *f*(H); ST = *f*(D); ST = *f*(H); BR = *f*(D); BR = *f*(H); ND = *f*(D); ND = *f*(H); RT = *f*(D); RT = *f*(H)). Also, the observed overestimation of t-scores was calculated for each of the 10 models for each dataset, using Eq 8.

### 2.4. Detecting the violation of independence assumption in linear models

The independence of residuals in linear models is usually checked using Durbin-Watson statistic (*d*) for spatial autocorrelation [23]:

$$d=\frac{\sum_{i=2}^{N}{\left(\varepsilon_{i}-\varepsilon_{i-1}\right)}^{2}}{\sum_{i=1}^{N}{\varepsilon_{i}}^{2}}$$ (12)

Where *N* is the total number of observations (trees). The value of *d* ranges between 0 and 4. If *d* takes values between *dL* (lower critical value) and *dU* (upper critical value), then the test is inconclusive. If *d* < *dL* then the null hypothesis of zero autocorrelation is rejected (accepting the alternative hypothesis, i.e., autocorrelation is greater than zero). If *d* > *dU*, then the null hypothesis of zero autocorrelation is accepted [24].

### 2.5. Software

The data was analysed using R version 3.3.0, packages nlme [25] and lmtest [26].

## 3. Results and discussion

### 3.1. Using clustered data with biomass allometric models: Review of published research

The analyzed literature revealed that 84 papers out of a total of 102 (82%, see S2 Appendix) used a clustered sampling design, and out of these 84 papers only 17 (20%) addressed the clustered data structure in their statistical analysis. The rest of the papers (67 papers, representing 80%) used linear models, despite the clustered sampling design (S2 Appendix). The papers ignoring the effect of clustered data in allometric models could have reported biased results only if ICC values in these papers (unknown, as the value was not reported) are different than zero. Otherwise (i.e. if ICC values are zero), the observations can be considered independent and using LM is appropriate. Out of total number of reviewed papers, 66% could have reported biased results.

### 3.2. The consequences of ignoring the clustered data structure

#### 3.2.1 On parameter estimates

Although LM and MLM use different methods for parameter estimation, these methods were shown to produce relatively similar results in a wide range of conditions [17]. Therefore, ignoring the clustered structure generally produces unbiased parameter estimates [27], although less efficient [28]. However, small differences between these two methods (i.e. LM and MLM) could appear. These differences were shown to be generally negligible, being lower than ±1.5% [17]. Nevertheless, we observed larger slope differences in our empirical example, of up to 4.5% (see S1 Table). A potential anomaly in the structure of clustered data that could produce bias in parameter estimates is the systematic difference of allometric scaling (β in Eq 2) within- and between-stands. If the within-stand allometric scaling is systematically lower (or larger) than between-stand scaling, the overall slope of the model is affected, and could produce unrealistic parameter estimates. A solution would be to use a random intercept only instead of random intercept and slope model (as the random intercept model forces slope to be equal within and between-stands), or use the ‘within-stand centering’ when appropriate [15].

#### 3.2.2. On standard errors

**Fig 1:**
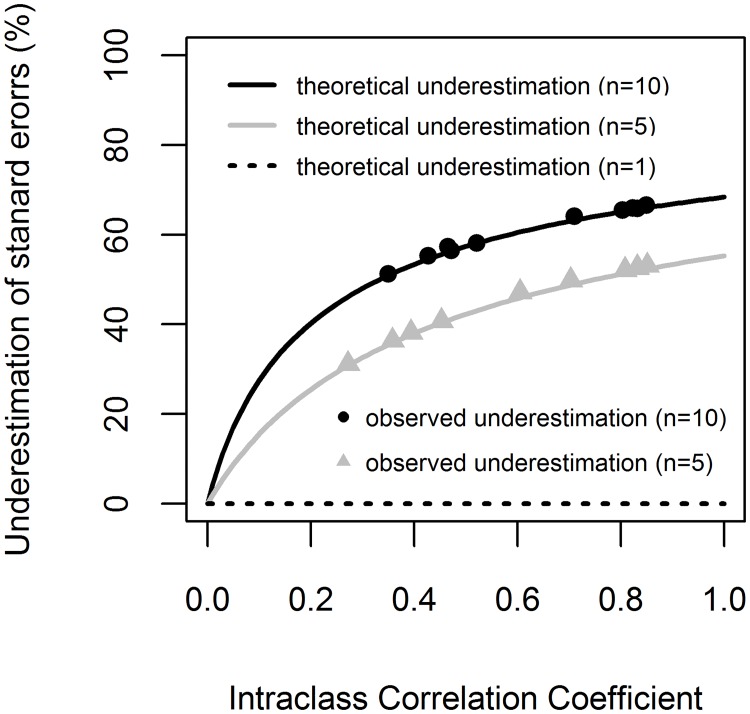
The underestimation of standard errors. The underestimation of standard errors by Intraclass Correlation Coefficient (ICC) for three cluster size values (*n* = 1, *n* = 5 and *n* = 10). The lines represent the theoretical standard error underestimation as resulted from Eq 7, and the symbols denote the observed underestimation resulted from Eq 6 (see S1 Table).

Our results showed that when ignoring the clustered data structure, the standard errors in logarithmic scale were underestimated by a rate that depended on both ICC and cluster size. In Fig 1 it can be observed that, the higher the ICC and cluster size, the higher the underestimation of standard errors. However, when ICC = 0 and *n* = 1, the underestimation is zero. Therefore, using LM with clustered data produces underestimated standard errors, when both ICC is greater than zero and cluster size is greater than one. For our empirical datasets, the observed underestimation of standard errors was greater than 31% when cluster size was 5 and greater than 51% when cluster size was 10 (S1 Table). These observed values fall on the theoretical lines (Fig 1), validating the assumptions of the study.

Testing whether the mean ICC observed in our empirical datasets equals zero (H_0_: ICC = 0), the null hypothesis was rejected at *P* < 0.0001 (*P* = 1.8e-11). Therefore, is extremely unlikely to observe these ICC values (see S2 Table) if ICC would have a mean of zero in the population. In return, the alternative hypothesis (H_A_: ICC > 0) was accepted. Heretofore, the ICC was never reported for allometric models. However, deriving the ICC values from reported random effects in biomass allometric studies [29,30], the results supported the alternative hypothesis (i.e. the derived ICC values were larger than 0.5) and not the null hypothesis.

#### 3.2.3. On confidence interval

**Fig 2:**
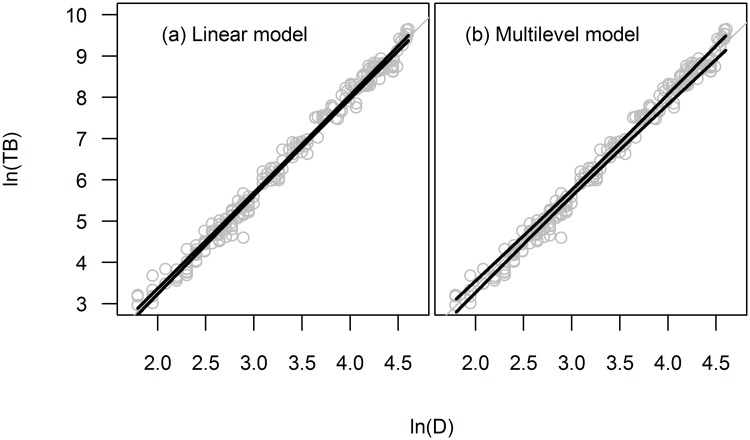
The 95% confidence interval in logarithmic scale. Presented for ln(TB) = *f* (ln(D)) when using dataset 2 (*n* = 10) for both linear (a) and multilevel model (b). (TB—total tree biomass; D—root collar diameter).

Uncertainty caused by allometric model selection is considered among the main sources of uncertainty in forest biomass estimation [31,32]. Producing allometric models with narrow confidence intervals (therefore with low uncertainty) is always preferred as long as that confidence interval was correctly estimated. Because of direct proportionality between standard errors and confidence intervals, the underestimation of confidence interval (of regression parameters) in logarithmic scale was similar to that of standard errors (Eq 7). Therefore, when ICC was larger than zero, the confidence intervals of parameters in logarithmic scale were underestimated as a function of ICC and cluster size (see Fig 1). Fig 2, shows that when clustered data structure was ignored, the 95% confidence interval of the model was narrower, producing overconfident biomass prediction.

In order to be used for biomass prediction, the linear allometric models require a back transformation, that includes the correction factor (Eq 11). The back transformation does not affect the slope (mean and confidence interval), but it does affect the intercept. After back transformation, the confidence interval of the intercept becomes asymmetrical. The reason is that standard error of the intercept resulted in logarithmic scale cannot be used as it is in the arithmetic scale. Confidence interval of the intercept should be therefore computed in logarithmic scale first and then the confidence interval bounds are back transformed. This transformation process produces lognormal asymmetry of the confidence interval of the intercept, yielding an asymmetric confidence interval of the model. The asymmetry depends on the length of its confidence interval in logarithmic scale. Therefore, for allometric models involving logarithmic transformation, the uncertainty is not symmetric to the mean.

#### 3.2.4. On null hypothesis test

**Fig 3:**
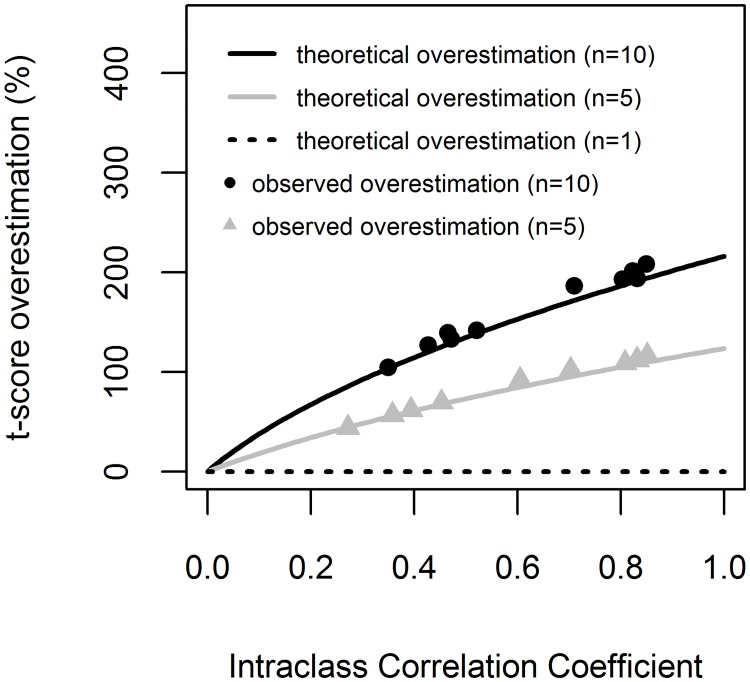
The overestimation of *t*-score when ignoring the clustering. The overestimation of *t*-score by Intraclass Correlation Coefficient (ICC) for three cluster sizes (*n* = 1, *n* = 5 and *n* = 10). The lines represent the theoretical *t*-score overestimation as resulted from Eq 10, and the symbols denote the observed overestimation as resulted from Eq 8 (for each dataset and each of the 10 models, see S2 Table).

Furthermore, the underestimation of standard errors affected the results of null hypothesis tests. Testing the significance of independent variable to predict biomass, involves the use of *t*-test (to test if the slope is different from zero). The *t*-score shows the evidence against null hypothesis (based on which the *P*-value is calculated). The false evidence against null hypothesis in *t*-test was removed by using the multilevel model. Therefore, LM showed overestimated evidence against null hypothesis in *t*-test (Fig 3).

The overestimation of *t*-score increased with ICC and with cluster size, *n* (Fig 3). The observed overestimation was greater than 46% when cluster size was 5 and greater than 108% when cluster size was 10 (S2 Table). It followed well the theoretical overestimation resulted from Eq 10. However, the observed differences between *β*_*lm*_ and *β*_*mlm*_ (see S1 Table) contradicts our assumption that *β*_*lm*_ = *β*_*mlm*_, and therefore the observed overestimation was slightly larger compared to theoretical one (Eq 10).

Clustered data can create problems especially when the *t*-scores are close to critical *t*-scores. As diameter (or height) and biomass are usually highly correlated [33], using just one independent variable (diameter or height) to predict biomass does not create problems on significance testing (as the slope’s *t*-scores are typically large enough not to concern). However, testing the slope significance of additional continuous independent variables, e.g. crown diameter [34], wood specific gravity [4], the *t*-scores could take values that are close to critical *t*-scores. In this case, the null hypothesis can be rejected although it might actually be true, resulting in an inflated type I error. Analysis of covariance (ANCOVA) which is often used to demonstrate differences between groups, is affected by clustering the same way as *t*-test, when two groups are involved.

### 3.3. Detecting the autocorrelation of residuals due to clustering

**Fig 4:**
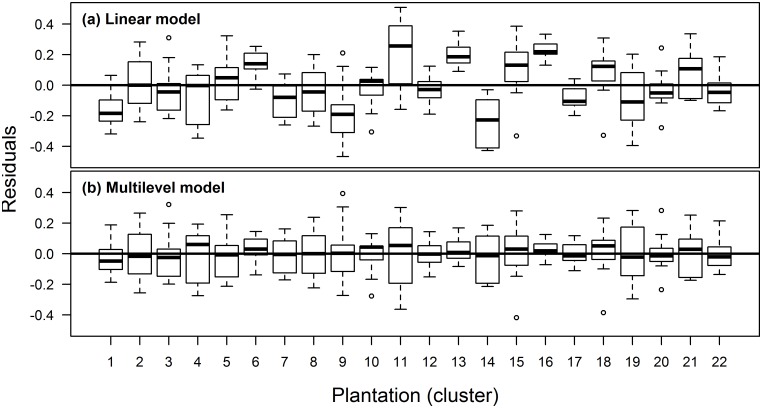
The residuals of TB = *f*(D) model (for dataset 2). Resulting from linear model (a) and multilevel model (b). Each boxplot describes the residuals within a cluster (10 residuals in each boxplot). (TB—total tree biomass; D—root collar diameter).

When multiple trees are sampled from multiple forest stands, each stand may induce a distinct pattern of biomass allocation to its own trees. As a result of this distinct pattern, the residuals in allometric models are not randomly located, but shifted in groups from the mean (regression line) (Fig 4a). This may create situations where consecutive residuals are located on the same side of regression line more often than usual, which produces autocorrelation. In Fig 4b is shown an example of how MLM approach can fix the autocorrelation.

Durbin-Watson statistic represents the traditional test for first-order spatial autocorrelation, and is often used in forestry studies (including biomass allometric models). Within this study, Durbin-Watson statistic (*d*) (Eq 12) showed values lower than 2.0 for all models (S2 Table). Based on the number of predictors, number of observations and significance level of 5%, the critical values were *dL* = 1.671 and *dU* = 1.707 for the first dataset, and *dL* = 1.770 and *dU* = 1.788 for the second dataset. Compared to these critical values, the actual *d* values were lower (showing positive autocorrelation), rejecting the null hypothesis of zero autocorrelation for all models. As a result, all LMs, without exception, have violated the independence assumption. Therefore, Durbin-Watson test has successfully detected the autocorrelation due to clustered structure of the data. However, Durbin-Watson test has limitations. When *d* > *dL* (*dL* is the lower critical Durbin-Watson value), the test is either inconclusive or shows lack of autocorrelation [23]. Therefore, as the autocorrelation is not significant, there is a temptation of ignoring the clustered structure and analyze the data using LM. This is not recommended, as Durbin-Watson test can fail to detect small ICC values (that corresponds to *d* > *dL*). Because the trees in the same forest stand are likely to be more similar, the test is likely to show positive autocorrelation only. Therefore, *d* interval of interest for allometric models ranges between 0.0 and 2.0 (instead of 4.0). Assuming a linear relationship between ICC and *d*, as ICC interval ranges between 0.0 and 1.0, ICC can be naively approximated as a function of *d*:

ICC=1.0-0.5×d (13)

**Fig 5:**
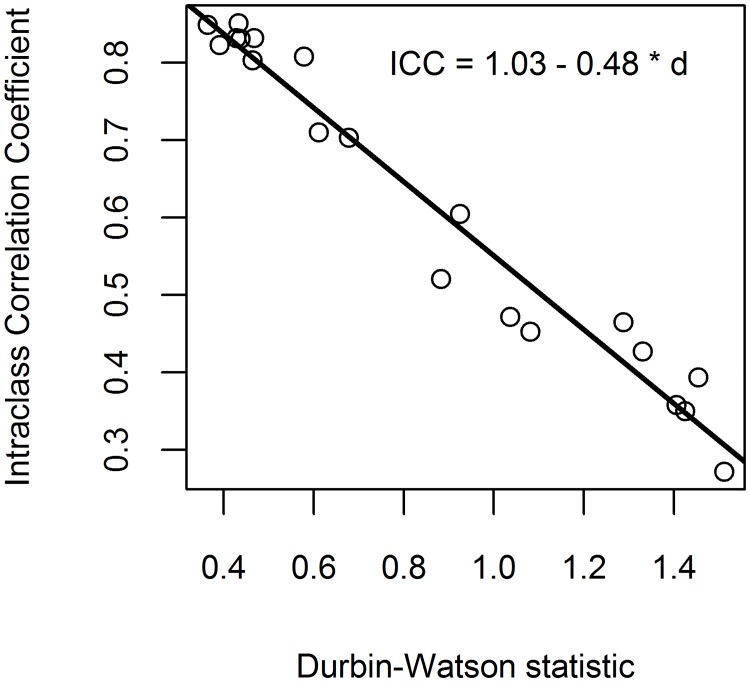
The observed relationship between Intraclass Correlation Coefficient (ICC) and Durbin-Watson statistic (*d*).

The observed relationship between ICC and *d* is displayed in Fig 5. Each of the 20 circles (observations) on the graph represent a model (10 models for each dataset: TB = *f*(D); TB = *f*(H); ST = *f*(D); ST = *f*(H); BR = *f*(D); BR = *f*(H); ND = *f*(D); ND = *f*(H); RT = *f*(D); RT = *f*(H)). Testing whether observed intercept and slope significantly differed from parameters of Eq 13, the results showed that both intercept and slope did not differ significantly from 1.0 (*P* = 0.098) and -0.5 (*P* = 0.156) respectively.

**Fig 6:**
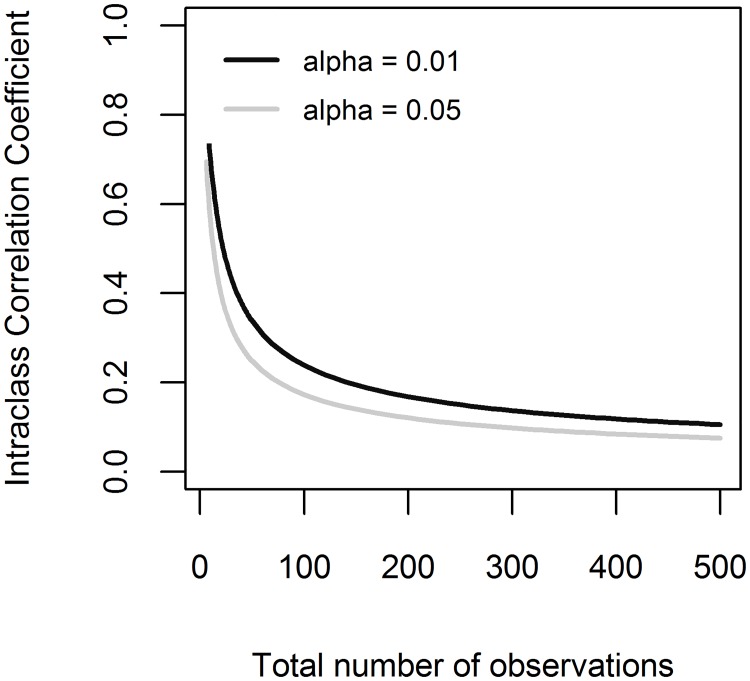
The Intraclass Correlation Coefficient (ICC) bounds under which Durbin-Watson test fails to detect the clustering. The ICC limits by total number of observations, shown for models with one predictor, for two significance levels (*α* = 0.01 and *α* = 0.05).

Using the *dL* values [24] in Eq 13, the ICC limits under which Durbin-Watson test fails to detect the autocorrelation due to clustering (for models with one predictor) are shown in Fig 6. Therefore, the ICC values under the curves are likely to be disregarded, as Durbin-Watson test shows lack of autocorrelation.

### 3.4. Sample size

#### 3.4.1. How many trees in each forest stand?

**Fig 7:**
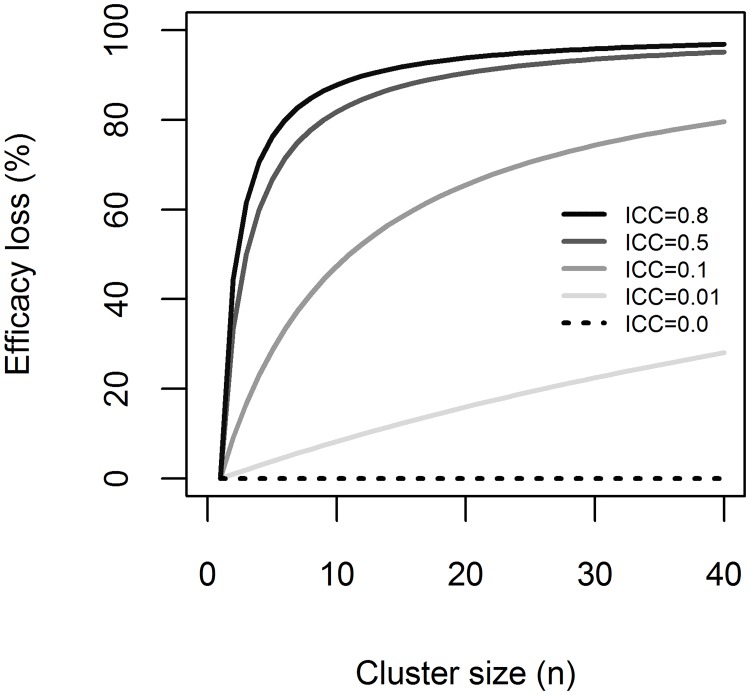
The efficacy loss by cluster size. Presented for five values of Intraclass Correlation Coefficient (ICC = 0, ICC = 0.01, ICC = 0.1, ICC = 0.5, ICC = 0.8). The efficacy loss was calculated using the function: *Efficacy loss (%) = (1–1/D*_*eff*_*) × 100*.

When planning a sampling design with clustered data, it is very important to know the ICC value. Although ICC is not usually known in advance, a rough estimation would tell whether a large or a small cluster size was appropriate. Sampling more than one tree per stand, engages a loss in observation efficacy (i.e. the loss of genuine information), which depends on ICC (Fig 7). When ICC > 0, every additional tree will bring less effective information into the model, compared to the previous sampled tree (e.g. the third sampled tree within a stand brings less effective information compared to the second sampled tree, and so on). In Fig 7, it can be observed that when ICC is very high, there is a sharp loss in efficacy.

However, a sufficiently large number of level 1 (trees) and level 2 (of forest stands) units are necessary in order to account for the variance within and between forest stands (which is needed to correctly estimate the ICC). A small number of trees in each forest stand (cluster size) can result in large bias in ICC estimation, which cannot be offset by increasing the number of forest stands [35]. Using just 2 trees per stand was shown to produce overestimated level 2 variance. However, sampling 5 trees or more in each forest stand was shown to produce valid and reliable estimates [27].

On the other hand, when ICC is high, a large number of trees in each forest stand is not cost-effective. Therefore, the perfect balance should be found between avoiding ICC bias and the loss of genuine information when increasing the number of trees sampled from each forest stand.

#### 3.4.2. How many forest stands?

To be assured of unbiased parameters, the number of forest stands should be higher than 50 when the models are used for inference [36]. However, when the parameter and their standard errors at level 1 are the principal interest (which is the case for most allometric models used for biomass prediction), the number of clusters appears less problematic. Even small numbers of forest stands and numbers of trees in each forest stand (10 forest stands and 5 trees per forest stand respectively) can offer unbiased estimates (parameters and standard errors) if ICC is higher than 0.1 [37]. Unequal numbers of trees in each forest stand, although shown to produce a loss in efficacy (which usually did not exceed 10%), can be compensated by increasing the number of forest stands by 11% [38].

### 3.5. Recommendations

It is highly recommended that residual autocorrelation is checked, using specific tests (e.g. Durbin-Watson), when there is no noticeable form of clustering (nesting) in the data. If the data is significantly auto-correlated, then ordinary least squares methods should not be used, due to increased risk of biased standard errors. The scientist should look for the cause of that autocorrelation and identify it before proceeding further with data analysis. Nevertheless, it is advisable that hierarchical models or other models that can account for the clustered data structure (e.g. robust standard errors, generalized least squares, cluster bootstrap, Bayesian hierarchical models [39,40]) are used when any form of clustering can be identified, even if the autocorrelation is not significant. This is because, even at very low ICC values (when Durbin-Watson test can indicate a lack of autocorrelation), the effect of clustering on standard errors can be substantial.

In multilevel models, the relationship between the dependent variable (e.g. biomass) and the independent variables (e.g. diameter, height) should be treated as a fixed effect. The random effect should be represented by the second (or higher) hierarchy within the data. This hierarchy could be represented by the forest stand, geographic region, tree species, position within canopy, or any other clustering factor that could alter the relationship between biomass and diameter (or height). Reporting the ICC value (or at least the random effects) with hierarchical allometric models is highly recommended.

We recommend adopting a sampling design based on large number of forest stands (at least 10) and large number of trees sampled from each forest stand (at least 5). However, if the number of forest stands is extremely low (e.g. lower than 4) as well as the number of trees in each forest stand (e.g. lower than 3), the resulting ICC could be highly imprecise [27,41]. In this case, for more accurate estimates, the standard errors resulting from LM may be manually adjusted by square root of design effect (Eq 4). The ICC value needed in Eq 4 could be deduced from random effects reported in the literature for similar species, forest stand characteristics and model type.

When deciding upon a cluster size, the key is to find the best compromise between the effort of measuring any additional tree within a forest stand, the ICC bias and the amount of genuine information that tree can bring to the model. Additionally, since the literature so far shows large variance attributed to forest stands [4,29,30], avoiding large cluster sizes is recommended, because it is likely that the effort invested in measuring additional trees within a forest stand will not be rewarded accordingly.

## 4. Conclusions

The reviewed published research shows that hierarchical approach is rarely used in biomass allometric models when the data is clustered. Our study demonstrates that, when ICC is different from zero, ignoring the clustering yields underestimated standard errors. Underestimation of standard errors has further consequences on model prediction and inference. The confidence intervals are also underestimated, resulting in overconfident tree biomass predictions. Additionally, the information against null hypothesis in *t*-test is overestimated, resulted in an inflated type I error, which may lead to potentially incorrect research conclusions. However, using first-order autocorrelation tests, such as the traditional Durbin-Watson statistic to detect the harmful effect of clustering could be misleading as the test may show lack of autocorrelation even though ICC is different from zero. Therefore, adopting a modelling approach that accounts for the hierarchical structure of the data is highly recommended when any form of clustering can be identified, even if the autocorrelation is not significant.

## Supporting information

S1 Appendix The location and characteristics of investigated plantations. (PDF)

S2 Appendix The list of reviewed papers. (PDF)

S1 Table The parameter estimates and their associated standard errors. Resulting from the Linear model and from the Multilevel model. (*SE*_*uα*_ is the underestimation of standard errors of the intercept and *SE*_*uβ*_ is the underestimation of standard errors of the slope).
(PDF)

S2 Table Durbin-Watson statistic, ICC and the *t*-score overestimation. (PDF)
