# Supplementary material for: Using clustered data to develop biomass allometric models: The consequences of ignoring the clustered data structure
Source: PLoS One. 2018 Aug 2;13(8):e0200123. doi: 10.1371/journal.pone.0200123 (PMC6071979; doi:10.1371/journal.pone.0200123)
Supplement: S1 Appendix — (PDF) [file pone.0200123.s001.pdf]

S2 Appendix. The location and characteristics of investigated plantations

Table 1. The characteristics of investigated plantations

Plantation no.	Latitude	Longitude	Altitude above sea level (m)	Exposition*	Slope (%)	Age (years)
1	47°35’15.4’’	25°00’41.6’’	1107	SE	10	4
2	45°31’98.1’’	25°57’55.1’’	1371	E	25	6
3	47°38’29.9’’	25°03’11.3’’	1024	SW	5	6
4	47°34’41.8’’	25°00’03.9’’	1048	SE	80	7
5	47°29’39.8’’	25°35’58.8’’	818	E	50	7
6	47°45’43.8’’	25°39’02.8’’	706	N	15	7
7	45°31’28.9’’	25°53’64.7’’	1350	N	16	7
8	45°33’46.9’’	25°54’82.7’’	941	W	3	8
9	45°31’06.0’’	25°35’15.8’’	989	NE	32	8
10	45°26’25.9’’	25°32’54.2"	937	NE	4	8
11	45°26’48.2’’	25°34’01.0’’	999	S	23	9
12	45°36’30.1’’	25°29’19.8’’	641	NW	12	9
13	45°27’07.5’’	25°34’15.0’’	1142	SW	15	9
14	45°27’29.3’’	25°34’01.9’’	961	SW	20	11
15	47°02’57.4’’	25°49’12.7’’	742	W	65	11
16	46°57’37.5’’	25°29’24.4’’	1114	NW	10	11
17	47°36’08.5’’	24°59’12.2’’	1122	S	20	12
18	47°11’17.6’’	26°03’18.0’’	688	SW	3	12
19	47°35’40.2’’	24°57’35.3’’	1563	S	90	12
20	45°26’36.2’’	25°32’56.4’’	942	E	5	13
21	46°04’37.0’’	26°21’38.0’’	900	NE	30	13
22	47°35’40.2’’	24°57’35.3’’	1085	SW	5	15

*S: South; N: North; E: East; W: West; SE: South-East; SW: South-West; NE: North-East; NW: North-West

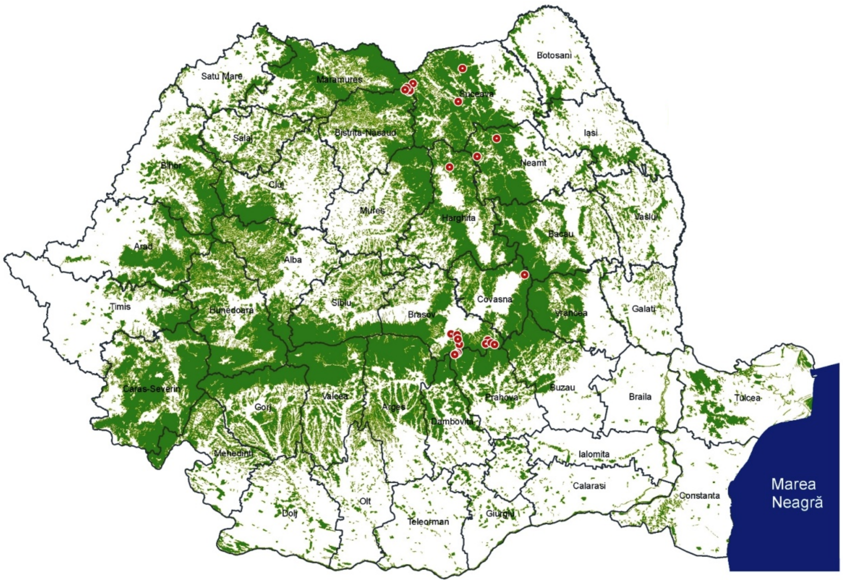

Figure 1. The location of the plantations (in red circles)
