# Supplementary material for: Using clustered data to develop biomass allometric models: The consequences of ignoring the clustered data structure
Source: PLoS One. 2018 Aug 2;13(8):e0200123. doi: 10.1371/journal.pone.0200123 (PMC6071979; doi:10.1371/journal.pone.0200123)
Supplement: S2 Appendix — (PDF) [file pone.0200123.s002.pdf]

S2 Appendix. The list of reviewed papers

Paper	No. of trees	No. of stands (or species)	Is the dataset independent?	Was the clustering effect included?	Details on sampling design
[1]	251	12	no	yes	251 trees; 9-31 trees from each site
[2]	96	14	no	no	14 species; 96 trees (multispecies model)
[3]	82	1	yes	n.a.	82 trees of P. serotina, sampled in winter and spring
[4]	4468	27	no	no	27 data sets
[5]	30	1	yes	n.a.	Not clear signs of clustering
[6]	18	1	yes	n.a.	18 stands; 1 tree per stand
[7]	45	6	no	no	83 (45 and 38) trees from 6 sites
[8]	115	6	no	no	6 sites; 115 trees in clusters
[9]	181	20	no	no	181 trees from 20 stands in 5 locations
[10]	406	39	no	no	406 trees, different species and sites
[11]	250	16	no	no	16 sites ; 6-30 trees from each site
[12]	200	20	no	no	200 trees from 20 stands
[13]	21	2	no	no	21 trees from 2 locations
[14]	160	31	no	no	160 trees from 31 sample plots
[15]	460	46 sp.	no	no	10 trees per species (multispecies model)
[16]	4004	58	no	no	4004 trees from 58 sites (standard errors not reported)
[17]	255	41	no	no	3 islands; 11-19 sites in each island
[18]	782	11	no	no	782 trees; general equation including all species
[19]	60	21	no	no	60 trees from 21 sites
[20]	20	1	yes	n.a.	20 trees per species
[21]	77	4	no	no	77 trees from 4 stands
[22]	20	1	yes	n.a.	Not clear if clustered
[23]	159	3	no	no	159 trees; 3 species; (multi-species model)
[23]	97	1	yes	n.a.	Not clear how many stands
[24]	850	5	no	no	5 sites, 850 trees
[25]	201	2	no	yes	201 trees; 2 countries
[26]	600	5	no	yes	5 locations; 120 trees from each location
[27]	478	3	no	no	20, 28, 30 plots from 3 locations; 478 trees from 9 species
[28]	60	5	no	no	60 trees in 5 regions
[29]	289	83	no	no	For each of three species, the trees were sampled from multiple plots (number of plots was lower than number of trees). See tables 1 and 2.
[30]	36	1	yes	n.a.	36 trees; (not clear signs of clustering)
[31]	164	13	no	yes	164 trees from 13 stands
[32]	138	47 sp.	no	no	138 trees; 47 species (multispecies model)
[33]	35	4	no	no	4 plantations; 35 trees (table 3)
[34]	120	4	no	no	120 trees; 30 per treatment
[35]	120	40	no	no	120 trees; from 40 plots
[36]	136	9	no	no	136 trees from 9 species; (family level model)

[37]	501	45	no	no	18 species; multiple plots (generic model)
[38]	25	2	no	no	25 trees from at least 2 stands (moderate and high saline)
[39]	110	41 sp	no	no	110 trees from 41 species (multispecies model)
[40]	222	26 sp.	no	no	222 trees from 2 regions and multiple tree species. Although mixed effects were used, the multispecies model does not account for the clustering of tree species.
[41]	11	1	yes	n.a.	11 trees from 1 pure stand
[42]	100	5	no	yes	5 locations; 16-26 trees from each location
[43]	24	1	yes	n.a.	24 stands; 1 tree per stand
[44]	74	4	no	no	74 trees from 4 forests
[45]	29	1	yes	n.a.	29 trees sampled from one forest stand
[46]	72	3	no	no	3 sites; 72 trees (12 for each diameter class)
[47]	72	3	no	no	3 sites; 72 trees
[48]	72	3	no	no	3 sites; 92 trees (20 for external validation)
[49]	48	3	no	no	48 trees; 29, 11, 8 for different species (multispecies model)
[50]	88	3	no	no	88 trees from 28 plots
[51]	320	16	no	yes	16 stands; 20 trees per stand
[52]	30	6	no	no	6 sites; 5 trees per site
[53]	2633	2	yes	n.a.	Not clear if clustered or not.
[54]	211	6	no	no	e.g. Model for clone 3478 using trees from 3 sites
[55]	64	17	no	no	64 trees from 17 forest stands (tab 1)
[56]	93	23	no	no	93 trees from 23 sites
[57]	639	5	no	no	5 sites; 180 + 459 trees (section 2.2.)
[58]	14	1	yes	n.a.	14 sample plots; 1 tree per plot
[59]	148	3	no	no	148 trees; 24 families; 3 sites
[60]	50	2	no	no	2 sites; 50 trees
[61]	162	2	no	no	2 locations; 162 shrubs
[62]	101	6	no	no	101 trees from 6 sites (13 species)
[63]	150	2	no	no	150 trees sampled from multiple sites and two origin types. Re-sprouting (59 trees) and originated from seeds (91 trees). Development of general model.
[64]	59	4 sp.	no	no	59 trees from 4 species. Multispecies model.
[65]	30	1	yes	n.a.	30 trees from 0.06 ha
[66]	39	1	yes	n.a.	39 trees ; from an 2.5 ha site
[67]	144	3	no	yes	3 sites; 144 clones
[68]	140	4	no	yes	5 clones; 4 populations
[69]	207	4	no	no	4 sites; (40, 47, 40, 40 trees); general model
[70]	40	20	no	no	40 trees from 20 farms
[71]	32	4	no	no	4 farms; 8 trees per farm
[72]	101	10	no	no	101 trees; 10 species (9-11 trees per species). Multispecies model
[73]	120	4	no	yes	4 sites; 10 species; 120 trees
[74]	3139	22	no	no	3139 individual trees and shrubs from 22 sites

[75]	2284	198	no	no	3384 trees from 198 sites
[76]	16	2	no	no	16 trees sampled from 2 states with different latitudes
[77]	26	2	no	no	26 trees from 2 locations and 2 species
[78]	122	4	no	yes	4 locations; 65 white spruce; 57 black spruce
[79]	75	3	no	no	3 sites; 27 plots; 75 trees
[80]	405	5	no	no	5 sites; 405 trees;
[81]	91	2	no	no	2 sites; 4 species (30, 30, 13, 18)
[82]	79	4	no	yes	79 trees; 4 treatments
[83]	36	5	no	no	36 trees from 5 stands
[84]	31	6	no	no	6 species; 55 plots; 31 trees (multispecies model)
[85]	67	17	no	yes	67 trees from 17 stands
[86]	80	2	no	yes	2 sites; 16 plots; 5 trees per plot
[87]	48	2	no	no	48 trees (12 trees per species and per site)
[88]	48	7	no	yes	7 sites; 48 trees; 4-8 trees from each stand
[89]	283	5	no	no	5 windbreaks of different age
[90]	120	8	no	no	120 trees: 8 stands; 5 clones; 3 blocks
[91]	57	4	no	no	4 species; 57 trees (multispecies equation, see table 2)
[92]	6	1	yes	n.a.	6 trees, one stand
[93]	230	12	no	yes	Clustered sampling (see table 1)
[94]	17	1	yes	n.a.	25 trees from one forest stand
[95]	147	41	no	no	147 trees from 41 species
[96]	143	8	no	no	8 sites; 143 trees sampled from all sites
[97]	604	2	no	yes	604 trees; 2 species; 2 origins
[98]	150	9	no	no	150 trees; from 8 provinces
[99]	64	1	yes	n.a.	64 individuals
[100]	80	1	yes	n.a.	Although the sampling area is large, no clear sign of clustering was shown
[101]	108	6	no	yes	108 trees from 6 stands
[102]	18	3	yes	n.a.	3 plantations; 6 trees in each plantation (models developed for each plantations)

n.a. – not applicable

References

1. Alfaro-Sánchez R, López-Serrano FR, Rubio E, Sánchez-Salguero R, Moya D, Hernández-Tecles E, et al. Response of biomass allocation patterns to thinning in Pinus halepensis differs under dry and semiarid Mediterranean climates. Ann For Sci. Springer Paris; 2015;72: 595–607. doi:10.1007/s13595-015-0480-y
2. Ali A, Xu M-S, Zhao Y-T, Zhang Q-Q, Zhou L-L, Yang X-D, et al. Allometric biomass equations for shrub and small tree species in subtropical China. Silva Fenn. 2015;49. doi:10.14214/sf.1275
3. Annighöfer P, Mölder I, Zerbe S, Kawaletz H, Terwei A, Ammer C. Biomass functions for the two alien tree species Prunus serotina Ehrh. and Robinia pseudoacacia L. in floodplain forests of Northern Italy. Eur J For Res. Springer-Verlag; 2012;131: 1619–1635. doi:10.1007/s10342-012-0629-2
4. Annighöfer P, Ameztegui A, Ammer C, Balandier P, Bartsch N, Bolte A, et al. Species-

specific and generic biomass equations for seedlings and saplings of European tree species. Eur J For Res. Springer Berlin Heidelberg; 2016;135: 313–329. doi:10.1007/s10342-016-0937-z

5. Aparecida De Mello A, Nutto L, Weber KS, Sanquetta CE, Luis J, De Matos M, et al. Individual Biomass and Carbon Equations for Mimosa scabrella Benth. (Bracatinga) in Southern Brazil. Silva Fenn. 2012;46. Available: http://www.metla.fi/silvafennica/full/sf46/sf463333.pdf
6. Battulga P, Tsogtbaatar J, Choimaa @bullet, @bullet D, Hauck M, Dulamsuren C, et al. Equations for estimating the above-ground biomass of Larix sibirica in the forest-steppe of Mongolia. J For Res. 2013;24: 431–437. doi:10.1007/s11676-013-0375-4
7. Beedy TL, Chanyenga TF, Akinnifesi FK, Sileshi GW, Nyoka BI, Gebrekirstos A. Allometric equations for estimating above-ground biomass and carbon stock in Faidherbia albida under contrasting management in Malawi. Agrofor Syst. Springer Netherlands; 2016;90: 1061–1076. doi:10.1007/s10457-015-9883-x
8. Beets PN, Kimberley MO, Oliver GR, Pearce SH, Graham JD, Brandon A. Allometric Equations for Estimating Carbon Stocks in Natural Forest in New Zealand. Forests. Molecular Diversity Preservation International; 2012;3: 818–839. doi:10.3390/f3030818
9. Bijak S, Zasada M, Bronisz A, Bronisz K, Czajkowski M, Ludwisiak Ł, et al. Estimating coarse roots biomass in young silver birch stands on post-agricultural lands in central Poland. Silva Fenn. 2013;47. doi:10.14214/sf.963
10. Blujdea VNB, Pilli R, Dutca I, Ciuvat L, Abrudan IV. Allometric biomass equations for young broadleaved trees in plantations in Romania. For Ecol Manage. 2012;264: 172–184. doi:10.1016/j.foreco.2011.09.042
11. Bouvet A, Nguyen-The N, Melun F. Nutrient concentration and allometric models for hybrid eucalyptus planted in France. Ann For Sci. Springer-Verlag; 2013;70: 251–260. doi:10.1007/s13595-012-0259-3
12. Bronisz K, Strub M, Cieszewski C, Bijak S, Bronisz A, Tomusiak R, et al. Empirical equations for estimating aboveground biomass of Betula pendula growing on former farmland in central Poland. Silva Fenn. 2016;50. doi:10.14214/sf.1559
13. Bulmer RH, Schwendenmann L, Lundquist CJ. Allometric Models for Estimating Aboveground Biomass, Carbon and Nitrogen Stocks in Temperate Avicennia marina Forests. Wetlands. Springer Netherlands; 2016;36: 841–848. doi:10.1007/s13157-016-0793-0
14. Chan N, Takeda S, Suzuki R, Yamamoto S. Establishment of allometric models and estimation of biomass recovery of swidden cultivation fallows in mixed deciduous forests of the Bago Mountains, Myanmar. For Ecol Manage. 2013;304: 427–436. doi:10.1016/j.foreco.2013.05.038
15. Chaturvedi RK, Raghubanshi AS. Aboveground biomass estimation of small diameter woody species of tropical dry forest. New For. Springer Netherlands; 2013;44: 509–519. doi:10.1007/s11056-012-9359-z
16. Chave J, Réjou-Méchain M, Búrquez A, Chidumayo E, Colgan MS, Delitti WBC, et al. Improved allometric models to estimate the aboveground biomass of tropical trees. Glob Chang Biol. 2014;20: 3177–3190. doi:10.1111/gcb.12629
17. Cienciala E, Centeio A, Blazek P, Cruz Gomes Soares M da, Russ R. Estimation of stem and tree level biomass models for Prosopis juliflora/pallida applicable to multi-stemmed tree species. Trees. Springer Berlin Heidelberg; 2013;27: 1061–1070. doi:10.1007/s00468-013-0857-1
18. Colgan MS, Swemmer T, Asner GP. Structural relationships between form factor, wood density, and biomass in African savanna woodlands. Trees. Springer Berlin Heidelberg; 2014;28: 91–102. doi:10.1007/s00468-013-0932-7
19. Copenhaver PE, Tinker DB. Stand density and age affect tree-level structural and functional characteristics of young, postfire lodgepole pine in Yellowstone National Park. For Ecol Manage. 2014;320: 138–148. doi:10.1016/j.foreco.2014.03.024
20. Cotillas M, Espelta JM, Sánchez-Costa E, Sabaté S. Aboveground and belowground biomass allocation patterns in two Mediterranean oaks with contrasting leaf habit: an insight into carbon stock in young oak coppices. Eur J For Res. Springer Berlin Heidelberg; 2016;135:

243–252. doi:10.1007/s10342-015-0932-9

21. Cutini A, Chianucci F, Manetti MC. Allometric relationships for volume and biomass for stone pine (Pinus pinea L.) in Italian coastal stands. http://www.sisef.it/iforest. SISEF - Italian Society of Silviculture and Forest Ecology; 2013;6: 331. doi:10.3832/IFOR0941-006
22. Da Silva F, Suwa R, Kajimoto T, Ishizuka M, Higuchi N, Kunert N. Allometric Equations for Estimating Biomass of Euterpe precatoria, the Most Abundant Palm Species in the Amazon. Forests. Multidisciplinary Digital Publishing Institute; 2015;6: 450–463. doi:10.3390/f6020450
23. Daryaei A, Sohrabi H. Additive biomass equations for small diameter trees of temperate mixed deciduous forests. http://dx.doi.org/101080/0282758120151089932. Taylor & Francis; 2015; doi:10.1080/02827581.2015.1089932
24. Das N. Allometric Modeling for Leaf Area and Leaf Biomass Estimation of Swietenia mahagoni in the North-eastern Region of Bangladesh. J For Environ Sci J Env Sci. 2014;30: 351–361. doi:10.7747/JFES.2014.30.4.351
25. de-Miguel S, Pukkala T, Assaf N, Shater Z. Intra-specific differences in allometric equations for aboveground biomass of eastern Mediterranean Pinus brutia. Ann For Sci. Springer Paris; 2014;71: 101–112. doi:10.1007/s13595-013-0334-4
26. Deb JC, Halim MA, Ahmed E. An allometric equation for estimating stem biomass of *Acacia auriculiformis* in the north-eastern region of Bangladesh. South For a J For Sci. Taylor & Francis Group ; 2012;74: 103–113. doi:10.2989/20702620.2012.701429
27. Dong L, Zhang L, Li F. Developing additive systems of biomass equations for nine hardwood species in Northeast China. Trees. Springer Berlin Heidelberg; 2015;29: 1149–1163. doi:10.1007/s00468-015-1196-1
28. Dong L, Zhang L, Li F. Allometry and partitioning of individual tree biomass and carbon of *Abies nephrolepis* Maxim in northeast China. Scand J For Res. Taylor & Francis; 2016;31: 399–411. doi:10.1080/02827581.2015.1060257
29. Dong L, Zhang L, Li F. Developing Two Additive Biomass Equations for Three Coniferous Plantation Species in Northeast China. Forests. Multidisciplinary Digital Publishing Institute; 2016;7: 136. doi:10.3390/f7070136
30. Durkaya B, Durkaya A, Makineci E, Ülküdür M. Estimation of above-ground biomass and sequestered carbon of Taurus Cedar (Cedrus libani L.) in Antalya, Turkey. http://www.sisef.it/iforest. SISEF - Italian Society of Silviculture and Forest Ecology; 2013;6: 278. doi:10.3832/IFOR0899-006
31. Elfving B, Ulvcrona KA, Egnell G. Biomass equations for lodgepole pine in northern Sweden. Can J For Res. NRC Research Press; 2017;47: 89–96. doi:10.1139/cjfr-2016-0131
32. Fayolle A, Doucet J-L, Gillet J-F, Bourland N, Lejeune P. Tree allometry in Central Africa: Testing the validity of pantropical multi-species allometric equations for estimating biomass and carbon stocks. For Ecol Manage. 2013;305: 29–37. doi:10.1016/j.foreco.2013.05.036
33. Fortier J, Truax B, Gagnon D, Lambert F. Plastic Allometry in Coarse Root Biomass of Mature Hybrid Poplar Plantations. BioEnergy Res. Springer US; 2015;8: 1691–1704. doi:10.1007/s12155-015-9621-2
34. García-Morote F, López-Serrano F, Martínez-García E, Andrés-Abellán M, Dadi T, Candel D, et al. Stem Biomass Production of Paulownia elongata × P. fortunei under Low Irrigation in a Semi-Arid Environment. Forests. Multidisciplinary Digital Publishing Institute; 2014;5: 2505–2520. doi:10.3390/f5102505
35. González-García M, Hevia A, Majada J, Barrio-Anta M. Above-ground biomass estimation at tree and stand level for short rotation plantations of Eucalyptus nitens (Deane & Maiden) Maiden in Northwest Spain. Biomass and Bioenergy. 2013;54: 147–157. doi:10.1016/j.biombioe.2013.03.019
36. Goodman RC, Phillips OL, del Castillo Torres D, Freitas L, Cortese ST, Monteagudo A, et al. Amazon palm biomass and allometry. For Ecol Manage. 2013;310: 994–1004. doi:10.1016/j.foreco.2013.09.045
37. Goussanou C, Guendehou S, Assogbadjo A, Kaire M, Sinsin B, Cuni-Sanchez A. Specific and generic stem biomass and volume models of tree species in a West African tropical semi-deciduous forest. Silva Fenn. 2016;50. doi:10.14214/sf.1474

38. Hossain M, Saha C, Rubaiot Abdullah SM, Saha S, Siddique MRH. Allometric biomass, nutrient and carbon stock models for Kandelia candel of the Sundarbans, Bangladesh. Trees. Springer Berlin Heidelberg; 2016;30: 709–717. doi:10.1007/s00468-015-1314-0
39. Huy B, Poudel KP, Temesgen H. Aboveground biomass equations for evergreen broadleaf forests in South Central Coastal ecoregion of Viet Nam: Selection of eco-regional or pantropical models. For Ecol Manage. 2016;376: 276–283. doi:10.1016/j.foreco.2016.06.031
40. Huy B, Poudel K, Kralicek K, Hung N, Khoa P, Phương V, et al. Allometric Equations for Estimating Tree Aboveground Biomass in Tropical Dipterocarp Forests of Vietnam. Forests. Multidisciplinary Digital Publishing Institute; 2016;7: 180. doi:10.3390/f7080180
41. Jia Q, Liu Q, Li J. Individual-based fine root biomass and its functional relationship with leaf for Pinus tabuliformis in northern China. Eur J For Res. Springer Berlin Heidelberg; 2015;134: 705–714. doi:10.1007/s10342-015-0884-0
42. Jiménez E, Vega JA, Fernández-Alonso JM, Vega-Nieva D, Álvarez-González JG, Ruiz-González AD. Allometric equations for estimating canopy fuel load and distribution of pole-size maritime pine trees in five Iberian provenances. Can J For Res. NRC Research Press; 2013;43: 149–158. doi:10.1139/cjfr-2012-0374
43. Johansson T. Biomass production of hybrid aspen growing on former farm land in Sweden. J For Res. Northeast Forestry University; 2013;24: 237–246. doi:10.1007/s11676-012-0305-x
44. Kachamba D, Eid T, Gobakken T. Above- and Belowground Biomass Models for Trees in the Miombo Woodlands of Malawi. Forests. Multidisciplinary Digital Publishing Institute; 2016;7: 38. doi:10.3390/f7020038
45. Kizha A, Han H-S. Predicting Aboveground Biomass in Second Growth Coast Redwood: Comparing Localized with Generic Allometric Models. Forests. Multidisciplinary Digital Publishing Institute; 2016;7: 96. doi:10.3390/f7050096
46. Kuyah S, Dietz J, Muthuri C, Jamnadass R, Mwangi P, Coe R, et al. Allometric equations for estimating biomass in agricultural landscapes: I. Aboveground biomass. Agric Ecosyst Environ. 2012;158: 216–224. doi:10.1016/j.agee.2012.05.011
47. Kuyah S, Dietz J, Muthuri C, Jamnadass R, Mwangi P, Coe R, et al. Allometric equations for estimating biomass in agricultural landscapes: II. Belowground biomass. Agric Ecosyst Environ. 2012;158: 225–234. doi:10.1016/j.agee.2012.05.010
48. Kuyah S, Muthuri C, Jamnadass R, Mwangi P, Neufeldt H, Dietz J. Crown area allometries for estimation of aboveground tree biomass in agricultural landscapes of western Kenya. Agrofor Syst. Springer Netherlands; 2012;86: 267–277. doi:10.1007/s10457-012-9529-1
49. Kuyah S, Dietz J, Muthuri C, van Noordwijk M, Neufeldt H. Allometry and partitioning of above- and below-ground biomass in farmed eucalyptus species dominant in Western Kenyan agricultural landscapes. Biomass and Bioenergy. 2013;55: 276–284. doi:10.1016/j.biombioe.2013.02.011
50. Kuyah S, Sileshi G, Rosenstock T. Allometric Models Based on Bayesian Frameworks Give Better Estimates of Aboveground Biomass in the Miombo Woodlands. Forests. Multidisciplinary Digital Publishing Institute; 2016;7: 13. doi:10.3390/f7020013
51. Leites LP, Zubizarreta-Gerendiain A, Robinson AP. Modeling mensurational relationships of plantation-grown loblolly pine (Pinus taeda L.) in Uruguay. For Ecol Manage. 2013;289: 455–462. doi:10.1016/j.foreco.2012.10.016
52. Lim H, Lee K-H, Lee KH, Park IH. Biomass expansion factors and allometric equations in an age sequence for Japanese cedar (*Cryptomeria japonica*) in southern Korea. J For Res. Springer Japan; 2013;18: 316–322. doi:10.1007/s10310-012-0353-2
53. Liu Y, Blanco JA, Wei X, Kang X, Wang W, Guo Y. Determining suitable selection cutting intensities based on long-term observations on aboveground forest carbon, growth, and stand structure in Changbai Mountain, Northeast China. Scand J For Res. Taylor & Francis; 2014;29: 436–454. doi:10.1080/02827581.2014.919352
54. Lupi C, Larocque G, DesRochers A, Labrecque M, Mosseler A, Major J, et al. Evaluating sampling designs and deriving biomass equations for young plantations of poplar and willow clones. Biomass and Bioenergy. 2015;83: 196–205. doi:10.1016/j.biombioe.2015.09.019
55. MacFarlane DW. A generalized tree component biomass model derived from principles of variable allometry. For Ecol Manage. 2015;354: 43–55. doi:10.1016/j.foreco.2015.06.038

56. Magalhães TM. Allometric equations for estimating belowground biomass of Androstachys johnsonii Prain. Carbon Balance Manag. 2015;16. doi:10.1186/s13021-015-0027-4
57. Makungwa S, Chittock A, Skole D, Kanyama-Phiri G, Woodhouse I. Allometry for Biomass Estimation in Jatropha Trees Planted as Boundary Hedge in Farmers' Fields. Forests. Multidisciplinary Digital Publishing Institute; 2013;4: 218–233. doi:10.3390/f4020218
58. Manolis EN, Zagas TD, Poravou CA, Zagas DT. Biomass assessment for sustainable bioenergy utilization in a Mediterranean forest ecosystem in northwest Greece. Ecol Eng. 2016;91: 537–544. doi:10.1016/j.ecoleng.2016.02.041
59. Manuri S, Brack C, Nugroho NP, Hergoualc'h K, Novita N, Dotzauer H, et al. Tree biomass equations for tropical peat swamp forest ecosystems in Indonesia. For Ecol Manage. 2014;334: 241–253. doi:10.1016/j.foreco.2014.08.031
60. Marziliano PA, Lafortezza R, Medicamento U, Lorusso L, Giannico V, Colangelo G, et al. Estimating belowground biomass and root/shoot ratio of Phillyrea latifolia L. in the Mediterranean forest landscapes. Ann For Sci. Springer Paris; 2015;72: 585–593. doi:10.1007/s13595-015-0486-5
61. Mason N, Beets P, Payton I, Burrows L, Holdaway R, Carswell F. Individual-Based Allometric Equations Accurately Measure Carbon Storage and Sequestration in Shrublands. Forests. Multidisciplinary Digital Publishing Institute; 2014;5: 309–324. doi:10.3390/f5020309
62. Mbow C, Verstraete MM, Sambou B, Diaw AT, Neufeldt H. Allometric models for aboveground biomass in dry savanna trees of the Sudan and Sudan-Guinean ecosystems of Southern Senegal. J For Res. Springer Japan; 2014;19: 340–347. doi:10.1007/s10310-013-0414-1
63. McNicol IM, Berry NJ, Bruun TB, Hergoualc'h K, Mertz O, de Neergaard A, et al. Development of allometric models for above and belowground biomass in swidden cultivation fallows of Northern Laos. For Ecol Manage. 2015;357: 104–116. doi:10.1016/j.foreco.2015.07.029
64. Mensah S, Veldtman R, Du Toit B, Glèlè Kakaï R, Seifert T. Aboveground Biomass and Carbon in a South African Mistbelt Forest and the Relationships with Tree Species Diversity and Forest Structures. Forests. Multidisciplinary Digital Publishing Institute; 2016;7: 79. doi:10.3390/f7040079
65. Morhart C, Sheppard J, Spiecker H. Above Ground Leafless Woody Biomass and Nutrient Content within Different Compartments of a P. maximowicii × P. trichocarpa Poplar Clone. Forests. Multidisciplinary Digital Publishing Institute; 2013;4: 471–487. doi:10.3390/f4020471
66. Morhart C, Sheppard JP, Schuler JK, Spiecker H. Above-ground woody biomass allocation and within tree carbon and nutrient distribution of wild cherry (Prunus avium L.) – a case study. For Ecosyst. 2016;3. doi:10.1186/s40663-016-0063-x
67. Mosseler A, Major JE, Labrecque M, Larocque GR. Allometric relationships in coppice biomass production for two North American willows (Salix spp.) across three different sites. For Ecol Manage. 2014;320: 190–196. doi:10.1016/j.foreco.2014.02.027
68. Mosseler A, Major JE, Larocque GR. Allometric relationships from coppice structure of seven North American willow (Salix) species. Biomass and Bioenergy. 2016;88: 97–105. doi:10.1016/j.biombioe.2016.03.025
69. Mugasha WA, Eid T, Bollandsås OM, Malimbwi RE, Chamshama SAO, Zahabu E, et al. Allometric models for prediction of above- and belowground biomass of trees in the miombo woodlands of Tanzania. For Ecol Manage. 2013;310: 87–101. doi:10.1016/j.foreco.2013.08.003
70. Negash M, Starr M, Kanninen M. Allometric equations for biomass estimation of Enset (Ensete ventricosum) grown in indigenous agroforestry systems in the Rift Valley escarpment of southern-eastern Ethiopia. Agrofor Syst. Springer Netherlands; 2013;87: 571–581. doi:10.1007/s10457-012-9577-6
71. Negash M, Starr M, Kanninen M, Berhe L. Allometric equations for estimating aboveground biomass of Coffea arabica L. grown in the Rift Valley escarpment of Ethiopia. Agrofor Syst. Springer Netherlands; 2013;87: 953–966. doi:10.1007/s10457-013-9611-3

72. Ngomanda A, Engone Obiang NL, Lebamba J, Moundounga Mavouroulou Q, Gomat H, Mankou GS, et al. Site-specific versus pantropical allometric equations: Which option to estimate the biomass of a moist central African forest? For Ecol Manage. 2014;312: 1–9. doi:10.1016/j.foreco.2013.10.029
73. Njana MA, Bollandsås OM, Eid T, Zahabu E, Malimbwi RE. Above- and belowground tree biomass models for three mangrove species in Tanzania: a nonlinear mixed effects modelling approach. Ann For Sci. Springer Paris; 2016;73: 353–369. doi:10.1007/s13595-015-0524-3
74. Paul KI, Roxburgh SH, England JR, Ritson P, Hobbs T, Brooksbank K, et al. Development and testing of allometric equations for estimating above-ground biomass of mixed-species environmental plantings. For Ecol Manage. 2013;310: 483–494. doi:10.1016/j.foreco.2013.08.054
75. Paul KI, Roxburgh SH, Ritson P, Brooksbank K, England JR, Larmour JS, et al. Testing allometric equations for prediction of above-ground biomass of mallee eucalypts in southern Australia. For Ecol Manage. 2013;310: 1005–1015. doi:10.1016/j.foreco.2013.09.040
76. Possu WB, Brandle JR, Domke GM, Schoeneberger M, Blankenship E. Estimating carbon storage in windbreak trees on U.S. agricultural lands. Agrofor Syst. Springer Netherlands; 2016;90: 889–904. doi:10.1007/s10457-016-9896-0
77. Poudel K, Temesgen H. Developing Biomass Equations for Western Hemlock and Red Alder Trees in Western Oregon Forests. Forests. Multidisciplinary Digital Publishing Institute; 2016;7: 88. doi:10.3390/f7040088
78. Power H, Schneider R, Berninger F. Understanding changes in black (Picea mariana) and white spruce (Picea glauca) foliage biomass and leaf area characteristics. Trees. Springer Berlin Heidelberg; 2014;28: 345–357. doi:10.1007/s00468-013-0953-2
79. Qi L, Liu X, Jiang Z, Yue X, Li Z, Fu J, et al. Combining diameter-distribution function with allometric equation in biomass estimates: a case study of Phyllostachys edulis forests in South Anhui, China. Agrofor Syst. Springer Netherlands; 2016;90: 1113–1121. doi:10.1007/s10457-015-9887-6
80. Rance SJ, Mendham DS, Cameron DM, Grove TS. An evaluation of the conical approximation as a generic model for estimating stem volume, biomass and nutrient content in young Eucalyptus plantations. New For. Springer Netherlands; 2012;43: 109–128. doi:10.1007/s11056-011-9269-5
81. Riofrío J, Herrero C, Grijalva J, Bravo F. Aboveground tree additive biomass models in Ecuadorian highland agroforestry systems. Biomass and Bioenergy. 2015;80: 252–259. doi:10.1016/j.biombioe.2015.05.026
82. Ritchie M, Zhang J, Hamilton T. Aboveground Tree Biomass for Pinus ponderosa in Northeastern California. Forests. Multidisciplinary Digital Publishing Institute; 2013;4: 179–196. doi:10.3390/f4010179
83. Samuelson LJ, Stokes TA, Butnor JR, Johnsen KH, Gonzalez-Benecke CA, Anderson P, et al. Ecosystem carbon stocks in *Pinus palustris* forests. Can J For Res. NRC Research Press; 2014;44: 476–486. doi:10.1139/cjfr-2013-0446
84. Sitoe A, Mandlate L, Guedes B. Biomass and Carbon Stocks of Sofala Bay Mangrove Forests. Forests. Multidisciplinary Digital Publishing Institute; 2014;5: 1967–1981. doi:10.3390/f5081967
85. Smith A, Granhus A, Astrup R. Functions for estimating belowground and whole tree biomass of birch in Norway. Scand J For Res. Taylor & Francis; 2016;31: 568–582. doi:10.1080/02827581.2016.1141232
86. Stark H, Nothdurft A, Bauhus J. Allometries for Widely Spaced Populus ssp. and Betula ssp. in Nurse Crop Systems. Forests. Multidisciplinary Digital Publishing Institute; 2013;4: 1003–1031. doi:10.3390/f4041003
87. Suchomel C, Pyttel P, Becker G, Bauhus J. Biomass equations for sessile oak (Quercus petraea (Matt.) Liebl.) and hornbeam (Carpinus betulus L.) in aged coppiced forests in southwest Germany. Biomass and Bioenergy. 2012;46: 722–730. doi:10.1016/j.biombioe.2012.06.021
88. Taeroe A, Nord-Larsen T, Stupak I, Raulund-Rasmussen K. Allometric Biomass, Biomass Expansion Factor and Wood Density Models for the OP42 Hybrid Poplar in Southern Scandinavia. BioEnergy Res. Springer US; 2015;8: 1332–1343. doi:10.1007/s12155-015-

9592-3
89. Tamang B, Andreu MG, Staudhammer CL, Rockwood DL, Jose S. Equations for estimating aboveground biomass of cadaghi (Corymbia torelliana) trees in farm windbreaks. Agrofor Syst. Springer Netherlands; 2012;86: 255–266. doi:10.1007/s10457-012-9490-z
90. Truax B, Gagnon D, Fortier J, Lambert F. Biomass and Volume Yield in Mature Hybrid Poplar Plantations on Temperate Abandoned Farmland. Forests. Multidisciplinary Digital Publishing Institute; 2014;5: 3107–3130. doi:10.3390/f5123107
91. Tumwebaze SB, Bevilacqua E, Briggs R, Volk T. Allometric biomass equations for tree species used in agroforestry systems in Uganda. Agrofor Syst. Springer Netherlands; 2013;87: 781–795. doi:10.1007/s10457-013-9596-y
92. Urban J, Čermák J, Ceulemans R. Above- and below-ground biomass, surface and volume, and stored water in a mature Scots pine stand. Eur J For Res. Springer Berlin Heidelberg; 2015;134: 61–74. doi:10.1007/s10342-014-0833-3
93. Vega-Nieva DJ, Valero E, Picos J, Jiménez E. Modeling the above and belowground biomass of planted and coppiced Eucalytpus globulus stands in NW Spain. Ann For Sci. Springer Paris; 2015;72: 967–980. doi:10.1007/s13595-015-0493-6
94. Verma A, Kaushal R, Alam NM, Mehta H, Chaturvedi OP, Mandal D, et al. Predictive models for biomass and carbon stocks estimation in Grewia optiva on degraded lands in western Himalaya. Agrofor Syst. Springer Netherlands; 2014;88: 895–905. doi:10.1007/s10457-014-9734-1
95. Xu Y, Zhang J, Franklin SB, Liang J, Ding P, Luo Y, et al. Improving allometry models to estimate the above- and belowground biomass of subtropical forest, China. Ecosphere. Ecological Society of America; 2015;6: art289. doi:10.1890/ES15-00198.1
96. Xue L, Lie G, Lu G, Shao Y. Allometric scaling among tree components in Pinus massoniana stands with different sites. Ecol Res. Springer Japan; 2013;28: 327–333. doi:10.1007/s11284-012-1021-x
97. Zeng W-S. Using nonlinear mixed model and dummy variable model approaches to develop origin-based individual tree biomass equations. Trees. Springer Berlin Heidelberg; 2015;29: 275–283. doi:10.1007/s00468-014-1112-0
98. Zeng W, Tang S. Modeling compatible single-tree aboveground biomass equations for masson pine (Pinus massoniana) in southern China. J For Res. Northeast Forestry University; 2012;23: 593–598. doi:10.1007/s11676-012-0299-4
99. Zhang C, Wang J, Zhao X, Xia F, Gadow K V. Sexual dimorphism in reproductive and vegetative allometry for two dioecious Rhamnus plants in north-eastern China. Eur J For Res. Springer-Verlag; 2012;131: 1287–1296. doi:10.1007/s10342-012-0598-5
100. Zhang C, Peng D-L, Huang G-S, Zeng W-S. Developing Aboveground Biomass Equations Both Compatible with Tree Volume Equations and Additive Systems for Single-Trees in Poplar Plantations in Jiangsu Province, China. Forests. Multidisciplinary Digital Publishing Institute; 2016;7: 32. doi:10.3390/f7020032
101. Zhang L, Deng X, Lei X, Xiang W, Peng C, Lei P, et al. Determining stem biomass of Pinus massoniana L. through variations in basic density. Forestry. Oxford University Press; 2012;85: 601–609. doi:10.1093/forestry/cps069
102. Zhou L, Shalom A-DD, Wu P, He Z, Liu C, Ma X. Biomass production, nutrient cycling and distribution in age-sequence Chinese fir (Cunninghamia lanceolate) plantations in subtropical China. J For Res. Northeast Forestry University; 2016;27: 357–368. doi:10.1007/s11676-015-0167-0
