# Supplementary material for: Using clustered data to develop biomass allometric models: The consequences of ignoring the clustered data structure
Source: PLoS One. 2018 Aug 2;13(8):e0200123. doi: 10.1371/journal.pone.0200123 (PMC6071979; doi:10.1371/journal.pone.0200123)
Supplement: S1 Table — Resulting from the Linear model and from the Multilevel model. (SEuα is the underestimation of standard errors of the intercept and SEuβ is the underestimation of standard errors of the slope). (PDF) [file pone.0200123.s003.pdf]

S1 Table. The parameter estimates and their associated standard errors. Resulting from the Linear model and from the Multilevel model; ($SE_{u\alpha}$ is the underestimation of standard errors of the intercept and $SE_{u\beta}$ is the underestimation of standard errors of the slope).

Cluster size (*n*)	Model	Linear model				Multilevel model				$SE_{u\alpha}$ (%)	$SE_{u\beta}$ (%)
		α	SE_α	β	SE_β	α	SE_α	β	SE_β		
n=5	TB=*f*(D)	-1.3980	0.0817	2.3523	0.0236	-1.3131	0.1285	2.3271	0.0371	36.4	36.4
	ST=*f*(D)	-2.7671	0.0820	2.3452	0.0237	-2.5672	0.1554	2.2859	0.0448	47.2	47.1
	BR=*f*(D)	-3.6113	0.1012	2.5567	0.0293	-3.5571	0.1471	2.5406	0.0425	31.2	31.1
	ND=*f*(D)	-2.3428	0.1100	2.3065	0.0318	-2.2377	0.1780	2.2753	0.0514	38.2	38.1
	RT=*f*(D)	-2.6440	0.1291	2.2204	0.0373	-2.5241	0.2179	2.1848	0.0629	40.8	40.7
	TB=*f*(H)	-6.5133	0.2662	2.5861	0.0523	-6.2088	0.5678	2.5258	0.1115	53.1	53.1
	ST=*f*(H)	-7.9741	0.2105	2.5996	0.0413	-7.8079	0.4446	2.5666	0.0873	52.7	52.7
	BR=*f*(H)	-9.1286	0.3186	2.8024	0.0625	-8.9776	0.6653	2.7725	0.1306	52.1	52.1
	ND=*f*(H)	-7.2853	0.3181	2.5213	0.0624	-7.0677	0.6712	2.4781	0.1317	52.6	52.6
	RT=*f*(H)	-7.4768	0.3019	2.4420	0.0593	-7.1965	0.6023	2.3864	0.1182	49.9	49.8
n=10	TB=*f*(D)	-1.4080	0.0585	2.3544	0.0169	-1.1447	0.1372	2.2762	0.0396	57.4	57.3
	ST=*f*(D)	-2.7695	0.0602	2.3470	0.0174	-2.5895	0.1438	2.2942	0.0416	58.1	58.2
	BR=*f*(D)	-3.6152	0.0738	2.5560	0.0213	-3.4536	0.1512	2.5079	0.0437	51.2	51.3
	ND=*f*(D)	-2.3455	0.0771	2.3069	0.0223	-2.0906	0.1723	2.2312	0.0498	55.3	55.2
	RT=*f*(D)	-2.6810	0.0875	2.2290	0.0253	-2.4451	0.2013	2.1589	0.0581	56.5	56.5
	TB=*f*(H)	-6.5164	0.1853	2.5831	0.0364	-5.9545	0.5544	2.4718	0.1088	66.6	66.5
	ST=*f*(H)	-7.9656	0.1489	2.5957	0.0292	-7.7406	0.4361	2.5511	0.0856	65.9	65.9
	BR=*f*(H)	-9.1138	0.2249	2.7950	0.0442	-8.7642	0.6520	2.7257	0.1279	65.5	65.4
	ND=*f*(H)	-7.2844	0.2194	2.5178	0.0431	-6.7988	0.6447	2.4216	0.1265	66.0	65.9
	RT=*f*(H)	-7.5163	0.2083	2.4454	0.0409	-7.0255	0.5806	2.3481	0.1139	64.1	64.1
