# Supplementary material for: Using clustered data to develop biomass allometric models: The consequences of ignoring the clustered data structure
Source: PLoS One. 2018 Aug 2;13(8):e0200123. doi: 10.1371/journal.pone.0200123 (PMC6071979; doi:10.1371/journal.pone.0200123)
Supplement: S2 Table — (PDF) [file pone.0200123.s004.pdf]

S2 Table. Durbin-Watson statistic, ICC and the *t*-score overestimation.

Cluster size (*n*)	Model	Linear model		Multilevel model				*t*-score of the slope resulted from:		t_{ovr} (%)
		Durbin Watson	AIC	Intercept variance	Residual variance	ICC	AIC	LM	MLM	
n=5	TB=*f*(D)	1.406	-43.6	0.0141	0.0253	0.358	-47.1	99.60	62.65	59.0
	ST=*f*(D)	0.925	-42.7	0.0256	0.0167	0.605	-75.1	98.88	50.94	94.1
	BR=*f*(D)	1.512	3.4	0.0162	0.0434	0.272	5.6	87.35	59.77	46.1
	ND=*f*(D)	1.454	21.7	0.0282	0.0433	0.394	13.3	72.52	44.25	63.9
	RT=*f*(D)	1.081	56.9	0.0447	0.0540	0.453	41.0	59.50	34.71	71.4
	TB=*f*(H)	0.433	106.7	0.1368	0.0240	0.851	-11.9	49.45	22.65	118.3
	ST=*f*(H)	0.468	55.1	0.0830	0.0168	0.832	-53.2	62.86	29.40	113.8
	BR=*f*(H)	0.578	146.2	0.1835	0.0437	0.808	46.9	44.78	21.22	111.0
	ND=*f*(H)	0.437	145.9	0.1890	0.0384	0.831	35.9	40.35	18.81	114.5
	RT=*f*(H)	0.678	134.4	0.1433	0.0604	0.703	71.1	41.18	20.17	104.2
n=10	TB=*f*(D)	1.288	-90.1	0.0202	0.0233	0.465	-137.1	139.69	57.42	143.3
	ST=*f*(D)	0.883	-75.5	0.0202	0.0185	0.521	-213.4	134.73	54.80	145.9
	BR=*f*(D)	1.425	13.9	0.0226	0.0419	0.350	-17.5	119.72	57.37	108.7
	ND=*f*(D)	1.331	33.3	0.0311	0.0418	0.427	-12.5	103.39	44.80	130.8
	RT=*f*(D)	1.036	89.1	0.0438	0.0489	0.472	25.1	88.00	37.12	137.1
	TB=*f*(H)	0.364	200.8	0.1360	0.0242	0.849	-93.7	70.99	22.72	212.5
	ST=*f*(H)	0.428	104.4	0.0832	0.0168	0.832	-175.3	88.81	29.81	197.9
	BR=*f*(H)	0.464	286.0	0.1830	0.0450	0.803	35.3	63.29	21.30	197.1
	ND=*f*(H)	0.391	275.1	0.1808	0.0389	0.823	6.2	58.45	19.14	205.4
	RT=*f*(H)	0.611	252.2	0.1397	0.0572	0.710	77.6	59.81	20.60	190.3
